# The Foreign Journals: Midwifery, and Diseases of Women and Children

**Published:** 1842-01

**Authors:** 


### MIDWIFERY, AND DISEASES OF WOMEN AND CHILDREN.

### *Account of a Case of Physometra.* By Dr. L. Ercoliani.

A young lady who had suffered much from hysteria and irregularities in menstruation, though otherwise she enjoyed good health, married when she had attained her twentieth year, and soon became pregnant. During pregnancy she suffered much from sickness and various nervous sensations, but her labour was attended by no accident, and for the first twenty-four hours after her delivery she appeared to be doing well. An attack of puerperal peritonitis then came on, which was treated actively by depletion and antiphlogistic remedies, and all the local symptoms subsided in the course of ten or twelve days. A degree of fever, however, still continued; for the removal of which the antiphlogistic plan was still persevered in, though, incessant vomiting having supervened, it was no longer possible to administer medicines by the mouth. Two months passed thus, the patient being occasionally somewhat better, but still labouring under a slow fever with exacerbations every evening, and becoming daily more and more emaciated. The uterus now began to enlarge, but without pain, or increase of the patient's general sufferings. Various remedies were tried, but without success, to allay the sickness; leeches were applied to the uterine region and a small dose of calomel had the effect of salivating the patient, whose illness had now lasted for four months. An attack of extremely severe pain in the abdomen now came on, lasting with brief intervals for two days and a half. During its continuance the patient shrieked with pain, and sometimes fainted from its severity. Each paroxysm was followed by the discharge of a small quantity of black fæces, but the sphincter ani seemed paralysed and injections thrown into the rectum returned immediately. At length an enema was retained, and the patient's sufferings almost immediately ceased.

Two days afterwards a considerable discharge of air and water took place from the uterus, and the patient was conscious of a constant escape of air between her thighs. The uterus diminished in size, the fever subsided and appetite returned. Within a month the lady was able to leave her room, but air still continued to issue from the uterus, and was often accompanied with a discharge of water. Water at length ceased to escape from the womb, but the escape of air continued for two years and a half, when the patient once more became pregnant. She had but very indifferent health while pregnant, but her labour was easy and her recovery rapid. During the flow of the lochia, however, the physometra, which had ceased during the last six months of pregnancy reappeared. From that time to the date of Dr. Ercoliani's report, two years had elapsed, during which time air still continued to escape from the uterus in various quantities, and at different intervals. This occurrence takes place at all times of the day, but especially in the morning; it is increased by the operation of purgatives, by mental excitement, or by sexual intercourse. The air which escapes is perfectly devoid of smell; the health of the lady is perfectly good, with the exception of occasional hysterical symptoms, and attacks of pain in the abdomen; which, however, are far less severe than those which she used to suffer before her second labour.

Dr. Ercoliani is disposed to think, that as a temporary occurrence the escape of air from the uterus, especially during the puerperal state, is by no means so rare as might be supposed from the little notice taken of it by most writers on the diseases of women.

*Annali Universali di Medicina. Decembre,* 1840.

---

*Account of a General Eruption of Vaccinia observed at the Hospital Cochin.*
By M. F. AUBRY.

THE instances are very rare in which an eruption of the pustules of vaccinia takes place at a distance from the spot where the lymph was inserted. The case related by M. Aubry is that of a female infant, who was vaccinated when a week old, three punctures being made in each arm. Vaccination was performed on July 3, 1841; on the 10th, virus was taken from her arm in order to inoculate other children. Several small papulæ were observed on her body on the 10th of July; and on the 15th eleven pustules, closely resembling those of the cow-pox, were seen on her abdomen and lower limbs. These pustules were of a flattened form, of a yellowish white colour, perfectly circular, with a slight depression in the centre, and surrounded by a bright red areola. The character of the eruption changed rapidly, and some of the pustules presented the brown scab, but this was not the case with all. The question of the nature of these pustules, however, was soon set at rest by vaccinating a child from them, when pustules were produced which underwent precisely those changes which are characteristic of chickenpox.

M. Cazenave denies that the pustules which occur on the body of patients who have been vaccinated are real vaccine pustules, except in those cases in which they have been produced by the person inoculating himself by scratching the skin of some part, with his fingers accidentally moistened with vaccine lymph from the parts where the puncture was made. But it cannot be supposed that in this case all the eleven pustules were thus produced, and we must therefore admit the possibility of a secondary eruption of vaccinia consequent on the absorption of the virus into the blood from the place where it was inserted. Nevertheless, it is probable that in the greaternumber of instances in which this has been supposed to occur, the eruptionsobserved were either varicella, or some varioloid eruption modified by vaccinia.

*Archives Générales de Médecine. Sept.* 1841.

*On the Performance of Tracheotomy in the last stage of Croup.* By M. PETEL, of Cateau.

THE object of this paper is to recommend the performance of the operation even in the last stage of the disease, a period when all other remedies are invariably useless. M. Petel has adopted it in six cases, in three of which the patients recovered, though five days had elapsed in one instance and ten in the other from the outbreak of the disease, and both patients were almost *in articulo mortis.* In every case warm water was thrown into the trachea after the operation several times, always with the effect of facilitating the discharge of mucosities. In one of the cases followed by recovery, a solution of 20 centigrammes of nitrate of silver to 4 grammes of water (about gr. iij. to ʒj.) was dropped into the air-passages, in the other calomel was blown into the trachea. The solution of nitrate of silver was likewise employed in two of the cases which terminated fatally. The subject of the fifth observation had been suffering from croup for eight days when tracheotomy was performed; the patient's life was saved by it, the wound in the trachea healed, and she survived six weeks after the operation, when she died suddenly in the night.

In performing the operation, M. Petel considers it to be inexpedient to delay opening the trachea on account of hemorrhage, provided the patient seems in a state of impending suffocation. His reasons for this advice are, 1st, that the hemorrhage is usually venous, and ceases almost immediately on the trachea being opened, while in the patient's semi-asphyxiated condition the colour of the blood affords but little clue as to its origin. If the hemorrhage be arterial, the vessel is usually of such small size that the bleeding can be checked by the application of nitrate of silver. And, 2d, blood which enters the bronchi does not, as is commonly supposed, occasion any serious consequences, but is expectorated as readily as the mucus which collects in the air tubes, or the fluids which may be injected into them.

*Journal des Connaissances Médico-Chirurgicales. Octobre,* 1841.

---

*On Myelitis Infantum.* By Dr. SCHLESIER.

DR. SCHLESIER has had two opportunities of observing this disease, both times in female children between three and four months old. After some days of general indisposition, the children became extremely feverish, and this fever presented frequent though irregular remissions and exacerbations. Children thus affected lie on the back, and cry if any one moves them from that position. Their dread of quitting it indeed is so great, that if taken in their mother's arms they refuse the breast, though they suck with avidity if the mother stoops over them so as to give them the breast while in the recumbent posture. The muscles of the neck and back now become stiff, the child anxiously avoids every movement. Muscular tremors come on, especially affecting the upper extremities, and the flexors more than the extensors. These convulsive movements gradually involve the whole body, twitchings of the face and of the upper extremities are constant, and the head is fixed perfectly stiff. An attack of convulsions with opisthotonos comes on, lasting five or six minutes, and returns after the lapse of six or eight hours. By degrees these attacks increase in severity and frequency; in the intervals the children lie in an apathetic state, whimpering rather than crying, but never obtaining any sleep. During the continuance of the disease, which lasts for some days, the bowels are costive, the urine scanty, and the fever considerable. At length copious sweats break out on the head, and during some fit more severe than the others the child dies.

Dr. Schlesier examined the cervical portion of the spinal cord of one child, the parents not allowing him to make a complete post-mortem inspection. He found considerable redness of the arachnoid and pia mater, and a copious exudation of lymph between the two membranes. The venous plexuses were loaded with blood, and the substance of the cord was softened and presented a dirty yellow colour.

*Medicinische Zeitung. Sept.* 8, 1841.

*On the Employment of the Plug, as a Means of Inducing Premature Labour.* By Dr. SCHÖLLER.

Two modes of producing premature labour are resorted to at the present day. Either the membranes are punctured, or a tent of sponge is introduced into the os uteri as recommended by Professor Kluge. The former method is liable to the objection of causing the too rapid escape of the liquor amnii, and thus endangering the life of the child when labour-pains are tardy in their occurrence. The second is often extremely difficult to accomplish, or even impossible when the os uteri is directed very much backwards, or when the patient has never before been pregnant.

Dr. Schöller was led to try the plug, from having observed that when used to suppress hemorrhage in cases of placenta presentation or of threatening abortion it had a considerable influence in exciting uterine action. He had observed also one case where its employment to restrain hemorrhage was followed by premature labour which it seemed to have occasioned. He recommends filling the upper part of the vagina with balls of charpie, which, for the sake of cleanliness should be renewed every twenty-four hours. The action of the uterus is usually very speedily excited, and when fully established, the plug may be withdrawn and secale cornutum administered to prevent the diminution of labour-pains. The finger may likewise be used to aid the dilatation of the os uteri, but great care must be taken to leave the membranes uninjured. Five cases are related in support of this practice. In four the child was born alive, but in the fifth, in which the breech presented, the infant was still-born. The average length of time which elapsed between the application of the plug and the birth of the child was five days and fourteen hours.

*Medicinische Zeitung. August* 18 and 25, 1841.

---

*Account of an Epidemic of Trismus Neonatorum, which occurred in the Lying-in Hospital at Stockholm in the year* 1834. By Prof. P. G. CEDERSCHJÖLD.

BEFORE the outbreak of the disease, sporadic cases of convulsive affections had been met with among the infants, putting on sometimes the form of trismus or tetanus, at other times differing but little from ordinary convulsions.

The commencement of the epidemic may be referred to the month of February, 1834, when one child was attacked by the disease. Three cases occurred in March, and two in April; but from the 8th of April to the 24th of May, the disease did not again show itself. From the 24th of May, however, to the 23d of June, 16 children were attacked by trismus, of whom 9 died. Another interval of 10 days elapsed without any fresh cases appearing; but between the 3d and 14th of July, three children died of trismus, and between the 14th and 21st, nine more infants were attacked and five died. After this time the cases of the disease became much less frequent, though it did not finally disappear until the 15th of November.

During the continuance of the epidemic 42 children were attacked by the disease, of whom 34 died, but it is probable that some of the cases reported as cured were not in reality instances of the disease, since no child recovered in whom the symptoms of trismus were at all well marked. Professor Cederschjold attributes the prevalence of the disease epidemically during the months of May, June, and July, to the great variableness of the weather ; but the circumstance of its not having occurred among the children of women delivered at their own homes, renders it probable that it was owing to some local cause.

The first symptoms usually appeared between the 4th and 6th day after birth, and the duration of the disease seldom exceeded two days. When attacked, a child became uneasy, cried aloud sometimes, and did not suck, though it was eager to take the breast. A slight convulsive movement was next observed about the lips, drawing apart the angles of the mouth. A convulsive seizure would next follow, sometimes resembling an ordinary convulsion, but at other times attended with trismus or tetanus. The alternation of trismus with general convulsions was frequently observed, but tetanus and convulsions were not nearly so often combined. Three periods might be observed in the convulsive attacks: the first was attended by symptoms of suffocation; during its continuance the whole body was stiff, and the face livid; convulsive movements, especially of the face and eyes, succeeded, and the livor disappeared; the third stage then came on, during which the child foamed at the mouth, and breathed difficultly and with a snoring sound. The attacks of tetanus occurred at longer intervals than those of convulsions; they were attended with opisthotonos, but seemed to exhaust the infants less than the convulsions.

Great congestion of the membranes and substance of the brain and spinal cord were found after death, and sometimes an extravasation of coagulated blood at the base of the cranium. The lungs were much collapsed, and often appeared to have been only incompletely inflated. The liver was usually much congested, and the gall-bladder distended.

The treatment consisted in the application of leeches to the nape of the neck, the administration of nauseating doses of emetics, and the use of antispasmodics and opium. Mild purgatives followed by antispasmodics seemed to be beneficial in some cases where premonitory symptoms of the disease had appeared, and laxatives, with the separation of the sick from the healthy, were employed as prophylactic measures.

*Neue Zeitschrift für Geburtskunde.* Band x. Heft iii. 1841.

